# The relationship of tobacco and alcohol use with ageing self-perceptions in older people in Ireland

**DOI:** 10.1186/s12889-016-3158-y

**Published:** 2016-07-22

**Authors:** Amanda Villiers-Tuthill, Antoinette Copley, Hannah McGee, Karen Morgan

**Affiliations:** Department of Psychology, Royal College of Surgeons in Ireland, Dublin, Ireland; PU-RCSI School of Medicine, Perdana University, Kuala Lumpur, Malaysia; Faculty of Medicine & Health Sciences, Royal College of Surgeons in Ireland, Dublin, Ireland

**Keywords:** Ageing perceptions, Health behaviours, Smoking, Alcohol, Older people

## Abstract

**Background:**

Health behaviour patterns in older groups, including tobacco and alcohol use, are key factors in chronic disease prevention. We explore ageing self-perceptions as motivating factors behind smoking and drinking alcohol in older adults, and the complex reasons why individuals engage harmfully in these behaviours.

**Methods:**

Cigarette and alcohol use was assessed in a large cross-sectional national sample aged 50 years and above from the Irish Longitudinal Study on Ageing (TILDA) (*n* = 6,576). The Brief Ageing Perceptions Questionnaire (BAPQ) assessed individual’s views of their own ageing across five domains. Study hypothesis that stronger beliefs on each of the BAPQ domains would be related to drinking and smoking was examined using multinomial logit models (MNLM). Regression parameter estimates for all variables were estimated relative risk ratios (RRR).

**Results:**

More women were non-drinkers (30 % vs. 20 %) and men displayed significantly higher alcohol use patterns. One in five older Irish adults was a current smoker (16.8 % of women, 17 % of men), and smoking and harmful drinking were strongly associated (*P* < .001). Some domains of ageing perceptions were significantly associated with harmful drinking and smoking. While the risk of being be harmful drinker decreased with stronger beliefs about the positive consequences of ageing (RRR 0.89), it increased with higher scores on both emotional representation and control positive domains.

Greater awareness of ageing and stronger emotional reaction to ageing increased likelihood of smoking. A greater sense of control over the outcomes of ageing was associated with increased risk of both harmful drinking (RRR control positive 1.16) and smoking (RRR control and consequences negative 1.25). This suggests optimistic bias in relation to perceived health risk from smoking and harmful drinking as a potential adverse effect of perceptions of control.

Risks of concurrent smoking and harmful drinking increased with chronic awareness of ageing (RRR 1.24), and negative emotional responses to it (RRR 1.21), and decreased with stronger perceptions of the positive consequences of ageing (RRR 0.85).

**Conclusions:**

The relationship between ageing perceptions, smoking and drinking is complex. Altering perceptions of ageing may be a useful intervention target aimed at facilitating engagement in preventative health behaviours in older people.

## Background

As life expectancy in developed countries increases, greater health and social needs associated with ageing are anticipated. The median age of the population in developed regions increased from 29 years in 1950 to 40 years in 2010, and is projected to reach 44 by 2050. Among those aged 60 years and over, the greatest increase has been in those aged 80 years and over [1]. While population ageing represents success in terms of advances in health care, it also poses considerable challenges as countries strive to meet the needs of their ageing populations and to bear the fiscal burden of multi-morbidity. Multi-morbidity is common in older people and is significantly associated with higher mortality, increased disability and functional decline [2].

Ageing is among the most important known risk factors for most chronic diseases [3]. Among leading causes of death of older adults are diseases of the circulatory system [4], diseases which are influenced by health behaviours across the lifecycle. An informed understanding of the factors that promote and inhibit the lifestyle choices made by older adults is important in the context of chronic disease prevention. This paper looks at the relationship between ageing self-perceptions and two of these health behaviours–tobacco and alcohol use.

Health behaviours can be defined as ‘behaviour patterns, actions and habits that relate to health maintenance, to health restoration and to health improvement’ [5]. The behaviours of particular relevance to chronic disease outcomes include drinking alcohol and smoking. Those aged over 75 years have been shown to be least likely to engage in preventive health behaviours, despite continued benefit of these behaviours throughout the life span [6–8]. In light of the expected epidemic of non-communicable diseases, it is important to assess the psychological antecedents of these risky health behaviours.

The older drinker differs from the younger drinker, physiologically, psychologically and socially, and so faces a different set of problems as a result of harmful drinking [9]. Older people are more vulnerable to the effects of alcohol, as it may interact differently with existing chronic conditions or with medications to adversely affect health outcomes [10]. Despite the psychosocial benefits associated with low to moderate alcohol consumption [11], studies have shown that harmful drinking negatively affects mental health [12] and that an individual’s drinking pattern is an independent risk factor for all-cause mortality and morbidity [13, 14]. Cigarette smoking is the leading cause of premature death among older adults. Half a million people in the European Union (EU) die from the effects of smoking each year with half of these deaths occurring in middle-aged or older adults [9]. Smoking cessation in older adults reduces the risks of cardiac disease, chronic obstructive pulmonary disease and smoking-related cancer mortality, reduces functional impairments and improves tolerance for exercise [15]. The reasons why people smoke and engage in harmful drinking have been studied extensively and include habit or addiction, relaxation, pleasure or enjoyment, boredom, as a weight control or social aid, and as a means of reducing negative affect [8, 16, 17]. Social, enhancement and coping motives for drinking have been shown to be most common among older adults [8]. Patterns of smoking and alcohol developed throughout life may form deeply engrained habits associated with multiple aspects of life, and are less dependent on physical functioning than other health behaviours such as being physically active.

A possible factor in these health behaviour choices might be self-perceptions of ageing. Ageing self-perceptions refers to the views that an individual holds regarding their own ageing and also refers to how the individual views themselves within the ageing process [18]. The formation of each individual’s perceptions and experiences of ageing is a dynamic process that pertains to self, social norms and their interplay, reflects the way an individual internalises social norms, and can change over time [19]. Ageing self-perceptions are significant correlates of well-being [20] and subjective [21] and functional health [18] as well as behavioural outcomes such as physical activity [22] and have been shown to influence survival and physical recovery from acute myocardial infarction [23, 24]. It has been shown that older adults with positive ageing self-perceptions are more likely to practice preventive health behaviours including eating a balanced diet, exercising, and following directions for taking prescribed medications over time [6], and that a perceived sense of control over one’s own age and ageing and a positive view of ageing can play an important role in determining the extent to which an individual is able or willing to change outcomes in relation to their own health [25, 26]. Similarly, negative self-perceptions of ageing have been shown to reduce health-related strategies of selection, optimization and compensation that are important for maintaining a healthy lifestyle [27]. However, the influence of ageing self-perceptions on specific health behaviours, namely smoking and alcohol consumption, has not been investigated.

Changing ageing self-perceptions may be an effective way of increasing participation in preventive health behaviours, thereby improving the overall health outcomes of older adults. This study aims to strengthen the evidence for the role of ageing self-perceptions as an important modifiable influence on chronic disease risk by elucidating their relationship with heretofore unexplored health behaviours. Analysis of data from the Irish Longitudinal Study on Ageing (TILDA) is undertaken with the aim of investigating the relationship between older adults’ self- perceptions of ageing and two specific health behaviours–smoking and drinking, taking account of important covariates, and describing patterns of these health behaviours in older Irish adults. The study hypothesis is that self-perceptions of ageing will be related to health behaviours.

## Methods

The TILDA study is the largest representative study of older adults in Ireland, providing a comprehensive overview of health, behavioural, social and economic data for those aged over fifty years living in Ireland. TILDA recruited a stratified clustered sample representative of the community living Irish population aged 50 years and over. Informed consent to participate in the study was obtained from all participants as part of the initial screening that preceded the study interview. Respondents were interviewed and also provided with a separate self-completion questionnaire containing questions on sensitive information, including alcohol intake and self-perceptions of ageing, which they were asked to complete and return by post. Wave one data was collected between 2009 and 2010. The response rate for the TILDA project was 60.2 % [28]. For the current study secondary analysis was conducted on data from a cross sectional sample of community-dwelling adults (age 50+ years) residing in Ireland, representing wave 1 TILDA respondents who had returned the self-completion questionnaire and for whom the sociodemographic and co-variables of interest were available. For variables with missing data below 5 % a system list-wise deletion of cases with missing data was adopted. As BAPQ variables had larger amounts of missing data, but were a key component of this study, missing data for these items was imputed using STATA 12.1 with multivariate imputation using chained equations (MI impute chained) with predictive mean matching. The final study sample included 6,576 respondents.

As this study involves only secondary analyses of the TILDA data set separate ethical approval for this study was not necessary. However, ethical approval for the TILDA project was sought and gained from the Faculty of Health Sciences Research Ethics Committee, Trinity College Dublin. Individuals’ views of their own ageing was assessed using the Brief Ageing Perceptions Questionnaire (BAPQ) [29]. This is a 17-item self-report questionnaire rating agreement with a range of statements on a 5-point Likert scale ranging from strongly disagree to strongly agree, to assess five domains (timeline chronic, consequences positive, consequences and control negative, control positive, and emotional representations). ‘Timeline chronic’ refers to the extent to which awareness of one’s age or ageing is chronic in nature, e.g. ‘I always classify myself as old’. ‘Consequences positive’ captures beliefs about the positive impact of ageing on one’s life across a variety of domains. ‘Consequences and control negative’ encompasses negative impacts of ageing on one’s life and a sense that it is not within the individual’s control to change these impacts. Conversely ‘control positive’ captures beliefs that the individual remains in control of managing their experience of aging. Lastly, ‘emotional representations’ includes items on the emotional response generated by aging, specifically negative emotions such as worry, anxiety, depression, fear, anger, and sadness [30].

The mean score is calculated for each subscale to yield a score of 1–5 for each of the domains with higher scores being indicative of greater endorsement of a specific perception. This questionnaire is a reduced version of the Ageing Perceptions Questionnaire (APQ) based on Leventhal’s self-regulation framework, which was validated by Barker and colleagues for the assessment of ageing perceptions, showing that ageing perceptions were independently associated with physical and psychological health indices [30]. The Brief APQ (BAPQ) was developed by Sexton and colleagues for ease of use in large population studies [29].

Alcohol was assessed using the Alcohol Use Disorders Identification Test (AUDIT-C), which includes questions on drinking frequency, quantity and binge drinking [31]. Current drinking patterns were then categorised into three groups, non-drinker, moderate drinker (those who stayed within the recommended weekly or daily safe limits or who stayed within the suggested number of alcohol-free days) and harmful drinker (those who consumed over the recommended daily or weekly limits or who consumed alcohol on more than four days per week), based on these questions and using guidelines for safe drinking set down by the Irish Department of Health (DOH). Low risk weekly guidelines for adults from the period 18 October 2009 to February 2011 when TILDA data was gathered, were up to 14 standard drinks in a week for women, and up to 21 standard drinks in a week for men, with at least 2 alcohol free days. A standard drink is a pub measure of spirits (35.5 ml), a small glass of wine (12.5 % volume), a half pint of normal beer, or an alcopop (275 ml bottle). In 2012 the recommended low risk guidelines for adults were reduced to 11 standard drinks in a week for women and up to 17 standard drinks in a week for men [32].

Questions about smoking included whether the respondent had ever smoked, whether they currently smoked (coded ‘Yes’ if the respondent had smoked in the past three months) and for former smokers the number of years they had smoked for and when they stopped, as well as the type (cigarettes, pipe, cigars or cigarillos) and quantity (number of cigarettes/pipes/cigars or cigarillos) of tobacco product used. Current smokers were defined as someone who reported having smoked consistently for one year (self-report of ‘number of years you smoked altogether’) and who smoked at the time the survey was carried out. Former smokers were defined as individuals who reported having smoked consistently for at least a one year period and who did not smoke at the time of the survey. The remaining participants were defined as never smokers.

Self-rated health was measured using five response options coded as: 1) excellent; 2) very good; 3) good; 4) fair; or 5) poor. Depression was assessed using the 20 item Centre for Epidemiologic Studies Depression Scale (CES-D), a self-administered scale measuring the frequency of major components of depressive symptomatology, including depressive mood, feelings of guilt and worthlessness, psychomotor retardation, loss of appetite and sleep disturbance [33]. Univariate analyses were used to calculate baseline demographics for the study sample and ageing self-perceptions. Demographic variables were compared using chi-square analyses and analyses of variance (ANOVA), as appropriate. To examine the study hypotheses that strong beliefs on each of the BAPQ domains would be related to drinking and smoking behaviours, multinomial logit models (MNLM) were fitted using each of the five domains on the BAPQ as the main independent variables. Firstly drinking category was taken as the dependent variable with non-drinkers being the reference group, secondly smoking status was the dependent variable with never smokers the reference group, and finally number of unhealthy behaviours was the dependent variable with ‘neither smokes nor drinks to excess’ as the reference group. Covariates controlled for in MNLMs included age, gender, education, marital status, self-rated health, socio-economic status, depression, smoking status (or drinking status, or neither) and physical activity. Interaction terms for age and gender were assessed and where necessary controlled for. The regression parameter estimates for all variables were estimated relative risk ratios (RRR).

## Results

Prior to imputation and deletion of cases with missing data as described, missing data for alcohol intake (0 %), smoking behaviour (0.01 %) and sociodemographic variables (sex 0 %, age 0.14 %, marital status 0 %, education 0 %, Socio Economic Status (SES) 0.01 %, self-rated health (SRH) 0 %, depression 1.56 %) ranged from 0 to 1.56 %, while missing data for BAPQ domains ranged from 19.6 % to 21.4 %.

The majority of the sample fell in the 50 to 64 years age group (58 %). Almost three quarters, (73.1 %) had achieved at least secondary level education. The majority were married (71.6 %) although 10.5 % of men and 7.9 % of women had never married. SRH was reported as good/fair by just under half of the sample (49.5 %), with 46 % rating it as excellent/very good and 4.5 % rating it as poor. Severe depression was reported in 6.1 % men and 11.1 % women and moderate depression in 14.9 % men and 19.6 % women.

Most men and women stayed within recommended daily alcohol limits (95.1 % women and 91.1 % of men were within the national recommendations or 14 standard drinks for women and 21 standard drinks for men). Women were more likely to be non-drinkers (29.5 % women vs. 20.4 % men) while men displayed significantly higher alcohol use patterns across all other frequencies of drinking. The highest proportion of non-drinkers (43.4 %) was among the oldest age group (75+). However, approximately one third of participants were at risk either because of drinking to excess in a binge drinking episode (71.7 % drank 3 standard drinks per day or less) or because they exceeded the weekly drinking limits (4.9 % women and 9.0 % men), see Table 1.

**Table 1:** Sociodemographic and health variables by drinking and smoking (*n* = 6576)

	Total population	Drinking behaviour			Smoking		
		Non drinker	Moderate drinker	Harmful drinker	Never	Former	Current
N (%)	6576	1666 (25.3)	2948 (44.8)	1962 (29.8)	2914 (44.3)	2550 (38.8)	1112 (16.9)
Age, years mean (SD)	63.18 (8.98)	66.76 (9.2)	62.82 (8.6)	60.69 (8.2)	62.23 (9.0)	64.14 (9.0)	60.87 (8.4)
Gender % (n)*,^							
Male	45.7 (3006)	20.4 (612)	40.6 (1220)	39.1 (1174)	36.0 (1081)	47.0 (1414)	17.00 (511)
Female	54.3 (3570)	29.5 (1054)	48.4 (1728)	22.1 (788)	51.4 (1833)	31.8 (1136)	16.8 (601)
Education % (n)*,^							
Primary	26.9 (1768)	37.1 (656)	37.0 (655)	25.8 (457)	37.1 (656)	41.0 (724)	22.0 (388)
Secondary	57.6 (3791)	22.3 (847)	46.1 (1749)	31.5 (1195)	46.0 (1744)	37.1 (1408)	16.9 (639)
Tertiary	15.5 (1017)	16.0 (163)	53.5 (544)	30.5 (310)	50.5 (514)	41.1 (418)	8.4 (85)
Marital Status % (n)*,^							
Married	71.58 (4707)	22.8 (1073)	46.2 (2175)	31.0 (1459)	45.7 (2149)	39.1 (1838)	15.3 (720)
Never married	9.06 (596)	29.4 (175)	41.8 (249)	28.9 (172)	40.6 (242)	39.6 (236)	19.8 (118)
Separated/divorced	6.34 (417)	21.3 (89)	39.8 (166)	38.9 (162)	34.5 (144)	34.3 (143)	31.2 (130)
Widowed	13.0 (856)	38.4 (329)	41.8 (358)	19.7 (169)	44.3 (379)	38.9 (333)	16.8 (144)
Socio-economic % (n)*,^							
Social class 1-2	23.9 (1574)	16.4 (258)	52.1 (820)	31.5 (496)	45.6 (718)	43.2 (680)	11.2 (176)
Social class 3-4	21.20 (1394)	25.8 (359)	44.1 (615)	30.1 (420)	43.8 (610)	41.0 (571)	15.3 (213)
Social class 5-6	12.0 (787)	28.7 (226)	37.9 (298)	33.4 (263)	34.7 (273)	42.1 (331)	23.2 (183)
Farmers	6.0 (398)	35.9 (143)	42.5 (169)	21.6 (86)	53.0 (211)	35.9 (143)	11.1 (44)
Unclassified	36.85 (2423)	28.1 (680)	43.2 (1046	28.8 (697)	45.5 (1102)	34.0 (825)	20.5 (496)
Self-Rated Health % (n)*,^							
Excellent/Very good	46.1 (3029)	21.4 (649)	47.7 (1446)	30.8 (934)	49.8 (1509)	36.6 (1108)	13.6 (412)
Good/Fair	49.5 (3252)	27.8 (904)	43.1 (1403)	29.1 (945)	40.5 (1316)	40.6 (1319)	19.0 (617)
Poor	4.5 (295)	38.3 (113)	33.6 (99)	28.1 (83)	30.12 (89)	41.7 (123)	28.1 (83)
Depression % (n)^							
None/mild	73.8 (4778)	24.6 (1177)	45.7 (2182)	29.7 (1419)	46.0 (2200)	39.4 (1883)	14.6 (695)
Moderate	17.4 (1128)	26.1 (294)	43.7 (493)	30.2 (341)	41.9 (473)	38.2 (431)	19.9 (224)
Severe	8.8 (572)	28.3 (162)	41.1 (235)	30.6 (175)	36.0 (206)	35.5 (203)	28.5 (163)

*P <0.05 chi square test across drinking categories, ^*P* <0.05 chi square test across smoking categories

One in five (16.9 %) older Irish adults was a current smoker including 16.8 % of women and 17 % men. Smoking prevalence reduced with age, for both men and women. Drinking status was strongly associated with smoking status (*X*^2^ = 252.07, *p =* 0.000) with the greatest risk seen among current smokers who were almost twice as likely to be harmful drinkers compared to never smokers (41.4 % vs 20.7 %).

Mean scores for each of the BAPQ domains and their covariate relationships with sociodemographic and health behaviour variables are reported in Table 2.

**Table 2:** Socio-demographic & self-rated health variation in ageing self-perceptions (*n* = 6576)

Characteristics	Timeline-chronic	Consequences-positive	Emotional-representation	Control-positive	Consequences and control negative
Age years	*P* < .001	*P* < .001	*P* < .001	*P* < .001	*P* < .001
ANOVA *p* value for F test					
50-64	2.30 (0.81)	3.85 (0.67)	2.25 (0.79)	4.00 (0.66)	3.15 (0.42)
65-74	2.50 (0.87)	3.72 (0.73)	2.29 (0.78)	3.92 (0.65)	3.08 (0.43)
75 & over	2.99 (0.91)	3.67 (0.73)	2.43 (0.84)	3.81 (0.68)	2.94 (0.43)
Sex	*P* < .001	*P* < .001	*P* < .001	*p =* .0161	*P* < .001
Male	2.54 (0.85)	3.75 (0.68)	2.24 (0.78)	3.93 (0.66)	3.08 (0.43)
Female	2.38 (0.89)	3.83 (0.72)	2.33 (0.81)	3.98 (.68)	3.12 (0.44)
Education	*P* < .001	*P* < .001	*P* < .001	*P* < .001	*P* < .001
Primary	2.71 (0.94)	3.71 (0.76)	2.39 (0.85)	3.84 (0.72)	3.07 (0.45)
Secondary	2.37 (0.83)	3.81 (0.67)	2.28 (0.78)	3.97 (0.63)	3.13 (0.43)
Tertiary	2.29 (0.79)	3.86 (0.65)	2.11 (0.73)	4.09 (0.64)	3.06 (0.41)
Marital status	*P* < .001	*p =* 0.2079	*P* < .001	*P* < .01	*P* < .001
Married	2.39 (0.84)	3.80 (0.69)	2.26 (0.78)	3.96 (0.65)	3.12 (0.43)
Never married	2.57 (0.90)	3.76 (0.70)	2.35 (0.81)	3.92 (0.65)	3.05 (0.42)
Sep/divorced	2.41 (0.86)	3.82 (0.70)	2.31 (0.82)	4.03 (0.66)	3.11 (0.42)
Widowed	2.73 (0.96)	3.76 (0.74)	2.39 (0.84)	3.89 (0.72)	3.03 (0.45)
Self-rated health	*P* < .001	*P* < .001	*P* < .001	*P* < .001	*P* < .001
Excellent/Very good	2.23 (0.80)	3.84 (0.68)	2.11 (0.72)	4.02 (0.66)	3.17 (0.43)
Good/Fair	2.61 (0.87)	3.76 (0.70)	2.42 (0.81)	3.90 (0.66)	3.05 (0.42)
Poor	2.93 (1.03)	3.70 (0.80)	2.69 (0.97)	3.83 (0.75)	2.93 (0.44)
Smoking Status	*P* < .001	*p =* 0.0025	*P* < .001	*p =* 0.0794	0.0241
Never	2.40 (0.87)	3.83 (0.69)	2.23 (0.78)	3.97 (0.67)	3.11 (0.44)
Former	2.46 (0.86)	3.76 (0.70)	2.30 (0.79)	3.95 (.0.65)	3.09 (0.43)
Current	2.56 (0.90)	3.90 (0.73)	2.40 (0.86)	3.93 (0.71)	3.13 (0.44)
Drinking behaviour	*P* < .001	*p =* 0.0953	0.3581	*P* < .001	*p =* 0.0170
Non drinker	2.57 (0.95)	3.78 (0.76)	2.30 (0.84)	3.87 (0.71)	3.08 (0.47)
Moderate	2.40 (0.85)	3.81 (0.68)	2.27 (0.78)	3.98 (0.66)	3.11 (0.43)
Harmful	2.42 (0.83)	3.77 (0.68)	2.30 (0.80)	3.89 (0.64)	3.11 (0.42)

*p* values for ANOVA of BAPQ domains across groups

Ageing perceptions were associated with drinking and smoking behaviour as outlined in Table 3. Controlling for age, gender, education, marital status, self-rated health, socio-economic status, depression, physical activity and smoking or drinking status two domains were significantly associated with relative risk of harmful drinking (consequences positive, emotional representation), and two with increased relative risk of current smoking (emotional representation, and control & consequences negative.

**Table 3:** Relative risk ratio for the association of ageing self- perceptions with drinking (*n* = 6510) and smoking behaviour (*n* = 6415), after correction for other covariates

Ageing Self Perception	RRR for association with drinking/smoking behaviour				RRR for concurrent smoking and harmful drinking
	Moderate drinker	Harmful drinker	Former smoker	Current smoker	
Timeline chronic	0.99	1.02	1.26^	1.3	1.18
	(0.92–1.07) ns	(0.93–1.11) ns	(0.76–2.10) ns	(0.67–2.52) ns	(1.03–1.33)*
Consequences positive	0.96	0.90	0.94	0.98	0.88
	(0.88–1.05) ns	(0.81–1.00)*	(0.87–1.02) ns	(0.88–1.09) ns	(0.76–1.02) ns
Emotional representation	1.11	1.18	1.07	1.13	1.05
	(1.02–1.21)*	(1.07–1.30)**	(0.99–1.15) ns	(1.03–1.25)*	(0.92–1.20) ns
Control positive	1.11	0.61^	0.97	0.93	0.86
	(1.01–1.22)*	(0.25–1.51) ns	(0.89–1.06) ns	(0.84–1.04) ns	(0.74–1.00) ns
Control & consequences negative	0.99	0.92	1.06	1.25	1.20
	(0.86–1.15) ns	(0.78–1.08) ns	(0.93–1.21) ns	(1.05–1.49) *	(0.93–1.53) ns

*p ≤ 0.05, **P < 0.01, *ns* not significant, *RRR* relative risk ratios (95 % Confidence Interval) ^interaction terms for age/sex were found to be significant and controlled for

Consequences positive was identified as a protective factor through its negative relationship with harmful drinking (RRR 0.90), while emotional representations increased the risk of harmful drinking (RRR 1.18). Individuals who scored higher on the consequences positive domain, indicating a greater awareness of the positive aspects of ageing, were 10 % less likely to engage in harmful drinking, and those who perceived stronger negative emotions in relation to ageing were 11 % more likely to be moderate drinkers and 18 % more likely to be harmful drinkers.

Emotional representations also increased the risk of being a current smoker such that those who felt more negative emotions in relation to ageing were 13 % more likely to be a current smoker. Those who felt a greater sense of the negative impacts of ageing on one's life and a sense that it is not within the individual’s control to change these impacts, were also at 25 % greater risk of being a current smoker.

Sex was found to be a significant confounder of the relationships between control positive and harmful drinking, and between timeline chronic and former smoking. Interaction terms were included in the model to control for this.

One ageing self-perception–emotional representation, is associated with increased risk of both drinking and smoking.

The only ageing perceptions significantly associated with concurrent smoking and harmful drinking behaviour was timeline chronic, as shown in Table 3.

## Discussion

This study aimed to assess the relationship between ageing self-perceptions and smoking and alcohol use, and describe the patterns of these health behaviours in older Irish adults. The study hypothesis that self-perceptions of ageing are related to health behaviours was confirmed.

### Drinking behaviour

Despite most respondents coming within recommended daily alcohol limits, it is important to recognise the significant number of older people at risk due to ‘binge drinking’ or exceeding the recommended weekly drinking limits. The authors of a previous Survey of Lifestyle, Attitudes and Nutrition in Ireland (SLÁN) report in Ireland noted that the majority of drinkers, particularly the middle-aged and older drinkers, were not aware that their drinking might be harming their health [34] and noted that many drinkers lack a clear understanding regarding safe drinking limits. Individuals who believe that moderate alcohol use is good for their health but who define moderate use above recommended guidelines, are more likely to be harmful drinkers [35]. Additionally the role alcohol has traditionally played in socialising in Ireland may contribute to these above recommended intakes.

We found that while some of the individual domains of ageing perceptions were implicated as risk factors for engaging in harmful health behaviours, one domain, consequences positive, was identified as a potential protective factor. This is in line with evidence that those with more positive self-perceptions of aging tend to practise more preventive health behaviours [6].

Pertaining to alcohol consumption, the relative risk ratios indicated that beliefs about positive consequences of ageing and a strong emotional representation of ageing were significantly associated with drinking status. Positive alcohol expectancies and positive drinking norms of friends or family mediate the relationship between ageing and drinking patterns, with those holding positive expectations about the role that alcohol plays in sustaining their moods and emotions more likely to report harmful drinking patterns compared with those with negative alcohol expectancies [24]. The results from this study suggest that perceptions of control over positive ageing experiences may similarly facilitate a positive relationship between ageing and drinking patterns, through unrealistic optimism about future outcomes, based on a lack of negative alcohol related experiences in the past.

Another important consideration is the scale used to measure these perceptions. The individual items comprising the subscale of ‘control positive’ are heavily loaded towards socialising, for example ‘The quality of my social life in later years depends on me’. In Ireland and specifically for this older population much social activity has traditionally taken place in places where alcohol is served. As such, respondents’ high scores on the control positive subscale may capture their perceptions or intentions around socialising at such venues, and hence influence the relationship of this variable with alcohol intake patterns.

There are opposing attitudes to alcohol in society–on one hand it is acknowledged as being harmful to health when taken in excess, but on the other it is often considered as a reward, an adjunct to sociability, and beneficial to health in moderate amounts although health benefits attributed to moderate intakes of alcohol may be misconstrued in the context of larger intakes. There is little differentiation made between moderate and harmful levels of drinking in these common associations, and as previously noted, drinkers may not be aware that their drinking is harming their health [34]. Stronger beliefs regarding control over positive aspects of ageing may result in drinking in order to achieve an increase in perceived benefits of alcohol.

### Smoking

Consistent with previous studies, this study found that smoking prevalence decreased with age, for both genders, however this may reflect higher mortality rates in smokers resulting in fewer smokers surviving to older age. Despite epidemiological studies recognising that the excess risk of premature death is halved in smokers who quit when they are between 65 and 70 years old [9], smoking cessation attempts are known to decline with age [36, 37] and older adults are more likely than younger smokers to underestimate the risks to their health [37]. The associations noted between smoking and harmful drinking are in keeping with evidence that unhealthy behaviours tend to occur in clusters [38] and the presence of other risky behaviours is a recognised barrier to quitting cigarettes.

In relation to smoking, the relative risk ratios indicated that, stronger emotional representation of the ageing process, stronger perceptions of control over negative aspects of ageing and awareness of the negative consequences were significantly associated with current smoking.

Those who felt negative emotions in relation to ageing were more likely to be a current smoker. These results, in tandem with the increased risk of harmful drinking among those with strong emotional representations of ageing, suggest that smoking and harmful drinking may be used as coping behaviours in this age group. Additionally, it has been noted that despite notable morbidity and mortality benefits to quitting smoking even at an advanced age, older adults with a belief that no health benefits could be gained from quitting at an advanced age are less likely to attempt to quit smoking or to relapse from an attempt [25].

The increased risk of being a current smoker among those with a greater sense of control over negative outcomes of ageing may be as a result of optimistic bias regarding the negative effects of smoking. Compared to drinking there tends not to be the same level of opposing attitudes to smoking in society, although individual smokers may attribute benefits such as stress release to their habit, it is largely acknowledged as an unhealthy behaviour with no associated health benefits at any level.

While perceptions of greater control over the experience of ageing (higher scores on control positive and control and consequences negative domains) may superficially sound positive, in this case we see increased risk of moderate drinking with greater perceptions of control over positive aspects of ageing and increased risk of smoking with increased perceptions of control and consequences negative, a sense of being aware of and able to control the negative aspects of ageing. The potential adverse effect of perceptions of control is the illusion of control [39], optimistic bias or false optimism. It has been acknowledged that people tend to display an unrealistic optimism in relation to perceived risk of occurrence of negative health outcomes [40]. Unrealistic optimism occurs when people perceive their own personal outcomes as being more positive than those of other people in similar circumstances [40].

Optimistic bias is not limited to any age, sex, educational or occupational group and tends to be introduced when people extrapolate from their past experience to estimate their future vulnerability, in other words the mistaken belief that if the problem has not yet appeared it will not appear in the future [41]. It is suggested that unrealistically optimistic or pessimistic risk perceptions may be associated with maladaptive health behaviours [42], for example adult smokers who were unrealistically optimistic about avoiding lung cancer were less likely to plan to quit smoking [43] and in a younger population unrealistic optimism regarding the effects of alcohol have been shown to predict increased negative alcohol related events [44]. This raises concern that similar negative consequences may befall older people who continue to smoke and drink harmfully in the belief that they will be able to control future ageing experiences.

### Concurrent smoking and drinking

When BAPQ domains were considered in relation to concurrent smoking and harmful drinking the timeline chronic domain was significantly associated with increased risk of this unhealthy behaviour pattern. Chronic awareness of ageing increased risk by 18 % further highlighting the important role of negative perceptions on the practical choices older people make.

Levels of moderate or severe depression, 21 % men and 30.7 % women, were high. The report on the first wave of TILDA notes that depression is common amongst older adults in Ireland with 10 % of the population reporting clinically significant depressive symptoms and a further 18 % reporting sub threshold levels of depression [28]. Depression is a risk factor for non-adherence to recommended health behaviour regimens [45] and has also been shown to have an inverse relationship with self-efficacy for health behaviours such as smoking and moderating alcohol intake [46], adding to the challenges often encountered in changing behaviour. While not a primary outcome measure in this study these figures warrant attention and should be further investigated as a potentially important influence on health behaviour choices among older adults.

This study used a nationally representative sample, allowing the implications of these results to be applied to the broader older population in Ireland. The recommended drinking limits in place at the time of data collection were high in comparison with international guidelines such as those published by the National Institute on Alcohol Abuse and Alcoholism in the United States, which recommend no more than seven drinks per week for women, and no more than fourteen drinks per week for men [47]. These differences should be borne in mind when considering the results of the current work in an international context. The potential bias caused by those who did not respond to questions about self-perceptions of ageing, and the subsequent imputation of missing data should be acknowledged as a limitation. Additionally, the data is cross-sectional preventing conclusions being drawn regarding directionality of the associations identified. Despite these limitations, this work highlights self-perceptions of ageing as a potential area for health behaviour intervention, warranting further attention.

## Conclusions

These results suggest that negative ageing perceptions such as negative emotional representations and chronic awareness of ageing are associated with increased health behaviour risk, while stronger perceptions of the positive consequences of ageing may reduce health behaviour risk, specifically harmful drinking. The relationships between an individual’s sense of control over the ageing process is complex as increased perceptions of control may, counterintuitively, increase the risk of harmful health behaviours.

Individuals’ perceptions regarding their age, positive and negative consequences of ageing, their control over the positive and negative aspects of ageing, and emotions relating to the ageing process all influence health behaviour choices. Those with positive ageing perceptions are less likely to engage in unhealthy patterns of concurrent alcohol use and smoking.

The cross sectional nature of this study must be counted as a limitation, and our results highlight the need for further longitudinal studies in this area to investigate the directionality of associations between ageing self-perceptions and health behaviours.

Adoption of healthy behaviours is influenced by personal attributes like perceptions, beliefs, values, expectations as well as emotional and affective states [5]. Older adults are vulnerable to engaging in risky behaviours such as harmful drinking and smoking for a variety of social and psychological reasons. A positive attitude to the ageing process has been identified as one mechanism for coping with radical life-altering situations. Similarly, positive or negative self-perceptions may account for some of the variability in the relationship between objective life conditions and subjective well-being.

Consideration of the mental and emotional processes that determine the actions required to support health highlights the role of self-perceptions in maintaining health. By examining health behaviours in the light of ageing self-perceptions we can better understand why some older adults engage in preventive health behaviours while others do not. The practical implications of these findings are that perceptions of the ageing experience may be targeted in interventions aimed at facilitating more adaptive outcomes or to moderate maladaptive outcomes associated with old age. Such interventions could be implemented at an individual level, at a societal level by means of public education, or at a clinical level by means of psychological intervention. In light of population ageing, such interventions could provide a novel approach to prospectively addressing chronic disease risk factors.

## Abbreviations

ANOVA, Analysis of Variance; APQ, Ageing Perceptions Questionnaire; AUDIT-C, Alcohol Use Disorders Identification Test; BAPQ, Brief Ageing Perceptions Questionnaire; CES-D, Centre for Epidemiologic Studies Depression Scale; DOH, Department of Health; EU, European Union; MNLM, Multinomial logit models; RRR, Relative risk ratios; SES, Socio Economic Status; SLÁN, Survey of Lifestyle, Attitudes and Nutrition in Ireland; SRH, Self-Rated Health; TILDA, The Irish Longitudinal Study on Ageing.
